# Overexpression of MicroRNA-16 Alleviates Atherosclerosis by Inhibition of Inflammatory Pathways

**DOI:** 10.1155/2020/8504238

**Published:** 2020-07-21

**Authors:** Manman Wang, Jiao Li, Jiageng Cai, Lijun Cheng, Xuewen Wang, Pengjuan Xu, Guangping Li, Xue Liang

**Affiliations:** ^1^Tianjin Key Laboratory of Ionic-Molecular Function of Cardiovascular Disease, Department of Cardiology, Tianjin Institute of Cardiology, The Second Hospital of Tianjin Medical University, Tianjin 300211, China; ^2^Department of Cardiology, Tianjin Union Medical Center, Nankai University Affiliated Hospital, Tianjin 300121, China; ^3^School of Integrative Medicine, Tianjin University of Traditional Chinese Medicine, Tianjin 301617, China

## Abstract

**Background:**

Our previous study demonstrated that the expression of miR-16 was downregulated in the cell and animal models of atherosclerosis (AS), a main contributor to coronary artery disease (CAD). Overexpression of miR-16 inhibited the formation of foam cells by exerting anti-inflammatory roles. These findings indicated miR-16 may be an anti-atherogenic and CAD miRNA. The goal of this study was to further validate the expression of miR-16 in CAD patients and explore its therapeutic roles in an AS animal model.

**Methods:**

A total of 40 CAD patients and 40 non-CAD people were prospectively registered in our study. The AS model was established in ApoE-/- mice fed a high-fat diet. The model mice were randomly treated with miR-16 agomiR (*n* = 10) or miR-negative control (*n* = 10). Hematoxylin-eosin staining was conducted for histopathological examination in thoracic aorta samples. ELISA and immunohistochemistry were performed to determine the expression levels of inflammatory factors (IL-6, TNF-*α*, MCP-1, IL-1*β*, IL-10, and TGF-*β*). qRT-PCR and western blotting were carried out to detect the mRNA and protein expression levels of PDCD4, miR-16, and mitogen-activated protein kinase pathway-related genes.

**Results:**

Compared with the normal control, miR-16 was downregulated in the plasma and peripheral blood mononuclear cell of CAD patients, and its expression level was negatively associated with IL-6 and the severity of CAD evaluated by the Gensini score, but positively related with IL-10. Injection of miR-16 agomiR in ApoE-/- mice reduced the formation of atherosclerotic plaque and suppressed the accumulation of proinflammatory factors (IL-6, TNF-*α*, MCP-1, and IL-1*β*) in the plasma and tissues but promoted the secretion of anti-inflammatory factors (IL-10 and TGF-*β*). Mechanism analysis showed overexpression of miR-16 might downregulate target mRNA PDCD4 and then activate p38 and ERK1/2, but inactivate the JNK pathway.

**Conclusions:**

Our findings suggest miR-16 may be a potential diagnostic biomarker and therapeutic target for atherosclerotic CAD.

## 1. Introduction

Coronary artery disease (CAD), which seriously endangers human health, is the leading cause of morbidity and mortality all around the world [1]. Atherosclerosis (AS), a complex, chronic, progressive disease of the arterial wall, is the major cause of CAD [2]. Several theories have been proposed to explain the pathogenesis of AS, including endothelial injury, lipid infiltration, smooth muscle cell cloning, platelet aggregation and thrombosis, inflammatory response, and oxidative stress [3, 4]. However, the detailed mechanisms have not been fully clarified, and effective diagnostic and therapeutic approaches remain limited.

MicroRNAs (miRNAs) are typically endogenous noncoding RNAs with intron properties which are composed of 22 nucleotides. Recently, accumulating evidence has revealed miRNAs play important roles in various physiological and pathological processes (such as inflammation, cell proliferation, and apoptosis) by binding to the 3′-untranslated region (UTR) of the targeted mRNAs and then inhibiting their translation or promoting their degradation [5]. Thus, dysregulation of miRNAs may be underlying mechanisms for the development and progression of AS and CAD, and targeting them may be a potential therapeutic strategy. This hypothesis has been confirmed by several scholars. For example, Su et al. [6] identified the expressions of both miR-181a-5p and miR-181a-3p were significantly downregulated in the aorta plaque and plasma of apolipoprotein-E-deficient (ApoE-/-) mice fed a high-fat diet and in the plasma of patients with CAD. Introduction of miR-181a-5p and miR-181a-3p to ApoE-/- mice reduced vascular inflammation and myeloid cell accumulation to vascular wall and retarded plaque formation via targeted regulating TAB2 and NEMO genes. Chen et al. [7] found the plasma miR-144 level was increased in CAD patients and associated with a higher SYNTAX score, an indicator to assess the extent and complexity of CAD. *In vitro* and *in vivo* assays showed overexpression of miR-144-3p resulted in accelerated pathological progression of AS by enhancing the expression of inflammatory factors and suppressing cholesterol efflux via downregulation of ATP-binding cassette transporter A1 (ABCA1) [8]. A decreased plasma miR-10a level was also correlated with high SYNTAX scores and serum tumor necrosis factor-*α* (TNF-*α*) and interleukin- (IL-) 6 levels in CAD patients [9, 10]. *In vivo* induction of miR-10a protected ApoE-/- mice from AS through inhibition of inflammatory cell infiltration through modulation of the downstream GATA6/vascular cell adhesion molecule- (VCAM-) 1 [11]. However, AS-related miRNAs remain rarely reported.

In 2016, our research team found the expression of miR-16 was reduced in the mice with AS and in the macrophage-derived foam cells. Transfection with miR-16 mimic suppressed the secretion and mRNA expression of proinflammatory TNF-*α* and IL-6, whereas it enhanced anti-inflammatory IL-10 in foam cells. The direct target of miR-16 was programmed cell death 4 (PDCD4) [12]. Furthermore, the study of Gu et al. [13] also reported lentiviral vector-mediated knockdown of miR-16 promoted Ang II-induced proliferation and migration in vascular smooth muscle cells. Microarray analysis and real-time PCR verified that miR-16 was significantly lower in the CAD patients than that in the non-CAD group [14]. Accordingly, we hypothesize miR-16 may be a potential diagnostic biomarker and a therapeutic target for atherosclerotic CAD. In this study, we aimed to further validate the expression of miR-16 in CAD patients because there was a controversial conclusion [15] and explore its therapeutic roles in an AS animal model which was not studied previously.

## 2. Materials and Methods

### 2.1. Study Population

To avoid the gender and estrogen effect, a total of 80 male patients with chest pain who underwent coronary angiography were prospectively enrolled in this study. The patients were equally divided into 2 study groups by coronary angiogram. The first was the control group consisting of patients who had chest pain, but CAD was excluded from by coronary angiogram. Segments were classified as having no significant stenosis (normal, or <50% lumen reduction). The second was the CAD group consisting of patients who had at least one diseased vessel (≥50% stenosis of luminal diameter). The inclusion criteria were as follows: (1) all patients who had typical chest pain and underwent coronary angiography, (2) no contraindication in the use of statin, and (3) no allergic history of a contrast agent. The exclusion criteria were as follows: (1) patients undergoing percutaneous coronary intervention or coronary artery bypass grafting, (2) left ventricular ejection fraction < 40%, (3) patients with heart valve disease, (4) patients with severe infection or malignant disease, (5) stroke, (6) patients with severe liver damage and renal dysfunction, and (7) statin allergy.

All angiograms were evaluated by two experienced interventional cardiologists. The severity of coronary artery lesions was assessed by the Gensini score [16]. This research obtained the approval of the Ethics Committee of the Second Hospital of Tianjin Medical University (KY2019K071). All patients were fully aware of the study process and signed the informed consent before this study.

### 2.2. Animal Experiments and Grouping

Twenty-two 4-6-week old male ApoE-/- mice (18-20 g) were available from the Animal Center of Tianjin Medical University (Tianjin, China). The mice were randomly divided into two groups and housed in circumstance of 22-23°C, 55-60% humidity under 12 h light-dark cycles, with free access to food and water. The animals were adapted for at least 7 days with a normal sterile diet before the experiment and then fed a high-fat diet in the following 20 weeks. Two mice were killed to assess whether the atherosclerotic model was successful. After successful modeling, the remaining 20 ApoE-/- mice were randomly divided into two groups: miR-16 agomiR group and miR-negative control group. The miR-16 agomiR (Ruibo Biotechnology Company, Guangzhou, China) was chemically modified and conjugated with cholesterol. A scrambled miR-16 agomiR (Ruibo Biotechnology Company, Guangzhou, China) synthesized as a negative control and miR-16 agomiR (10 nmol) conjugated with cholesterol and scrambled miR-16 agomiR in 0.1 ml PBS buffer were, respectively, injected into the tail vein of mice once every 5 days for 4 weeks. Animal experiments were approved by the Ethics Committee of Tianjin Medical University and were performed in accordance with National Institute of Health (NIH) Guide for the Care and Use of Laboratory Animals.

### 2.3. Sampling of Human Plasma and Peripheral Blood Mononuclear Cells (PBMCs)

Peripheral venous blood samples (5 mL) of all participants were collected into EDTA-coated tubes 2-4 h before coronary angiography. Partial blood samples (3 mL) were left naturally at normal atmospheric temperature for 30 min and then centrifuged at 4°C for 10 min at 1200 g to obtain platelet-poor plasma. The remaining blood samples were used for the separation of PBMCs using Ficoll-Hypaque. Following that, the plasma samples and monocytes were collected into RNase/DNase-free tubes and stored at -80°C for miRNA detection.

### 2.4. Measurement of Blood Biochemical Index

After 20 weeks of high-fat diet in mice, blood was taken from the heart of the mice after anesthesia and then centrifuged at a speed of 3000 r/min for 10 min to obtain plasma which was stored at -20°C later. Following that, the plasma of each group was concentrated and the levels of triglyceride (TG), total cholesterol (TC), and low-density lipoprotein cholesterol (LDL-C) and high-density lipoprotein cholesterol (HDL-C) and other lipid indicators (unit: mmol/L) were determined using an automated biochemical analyzer.

### 2.5. Measurement of Plasma Inflammatory Factors

The levels of inflammatory factors IL-6, TNF-*α*, monocyte chemotactic protein 1 (MCP-1), IL-1*β*, IL-10, and transforming growth factor-*β* (TGF-*β*) in the plasma were determined using the commercially available enzyme-linked immunosorbent assay (ELISA) kit (R&D Systems, Minneapolis, MN, USA) according to the manufacturer's protocols. The absorbance was read at 450 nm.

### 2.6. Histological and Immunohistochemical Analysis

The proximal segment of the thoracic aorta was fixed in 4% paraformaldehyde for 24 h at room temperature, then dehydrated and embedded in paraffin and cut into 5 *μ*m sections. The paraffin sections were baked for 1-2 h at 60°C and countersigned with hematoxylin-eosin (H&E) and immunohistochemical staining. The sections were immunostained with anti-MCP1 (Abcam, Cambridge, UK; ab25124, 1 : 200 dilution), anti-TNF-*α* (Abcam, Cambridge, UK; ab9739, 1 : 300 dilution), anti-IL-1*β* (Abcam; ab82558, 1 : 500 dilution), anti-IL-6 (Abcam; ab7737, 1 : 50 dilution), anti-IL-10 (Abcam; ab189392, 1 : 200 dilution), anti-TGF-*β* (Abcam; ab170874, 1 : 200 dilution), and anti-CD68 (a measure of macrophage load) [17] (Abcam; ab31630, 1 : 500 dilution). All images were taken at 100 and 200 magnifications under a light microscope (Olympus, Tokyo, Japan) and semiquantitatively measured using Image-Pro Plus software (version 6.0; Media Cybernetics).

### 2.7. Western Blotting

Total proteins were extracted from the thoracic aorta tissues using the radioimmunoprecipitation buffer lysis buffer (Roche, Mannheim, Germany). Total protein concentration was measured using the BCA Protein Assay Kit (Pierce, Rockford, USA) according to manufacturer's instructions. The same amount of total proteins were injected into the sample holes of 10% sodium dodecyl sulfonate- (SDS-) polyacrylamide gel electrophoresis (PAGE) and then electrophoretically transferred to polyvinylidene fluoride (PVDF) membranes (Millipore, Billerica, MA, USA). After being blocked with 5% skim milk for 1 h, the membranes were incubated overnight at 4°C with anti-p38 (Affinity Bioscience, USA; AF6456, 1 : 1000), anti-p-p38 (Affinity Bioscience, USA; AF4001, 1 : 1000), anti-JNK (Abcam; ab179461, 1 : 1000), anti-p-JNK, (Abcam; ab124956, 1 : 5000), anti-ERK (Affinity Bioscience, USA; AF0155, 1 : 5000), anti-p-ERK (Affinity Bioscience, USA; AF1015, 1 : 2000), and anti-*β*-actin (Abcam; ab8226, 1 : 5000). The membranes were then incubated with secondary antibodies, including goat anti-mouse IgG-H&L (HRP) (Abcam; ab205719, 1 : 10000) and goat anti-rabbit IgG-H&L (HRP) (Abcam; ab205718, 1 : 10000). Protein signals were assessed using enhanced chemiluminescence (ECL) western blot detection system (Millipore, Billerica, MA, USA) and densitometric analysis.

### 2.8. Quantitative Real-Time Polymerase Chain Reaction (qRT-PCR)

miRNA was extracted from blood plasma and PBMCs using miRcute Serum/plasma miRNA isolation kit (TianGen, Beijing, China). Total RNA was isolated from the thoracic aorta tissues using TRIzol® reagent (Invitrogen). miRNA or total RNA was reverse transcribed into cDNAs for 15 min at 42°C and 95°C for 3 min using FastQuant RT Kit (TianGen, Beijing, China). qRT-PCR was carried out to validate the expressions of miR-16 and PDCD4 using the SYBR Green PCR kit (TransGen, Beijing, China) according to the manufacturer's instructions. The PCR amplification was conducted by denaturation at 95°C for 10 min, followed by 40 cycles of 95°C for 30 sec, 58°C for 30 sec, and 72°C for 30 sec. The PCR was performed on Perkin-Elmer Gene Amp PCR system 2400 (Applied Biosystems, USA). U6 snRNA and glyceraldehyde 3-phosphate dehydrogenase (GAPDH) were used as an internal control for the expression of miRNA and mRNA, respectively. The primer sequences for reverse transcription and qRT-PCR are listed in Table 1. The relative expression levels of the genes were analyzed using the 2^-*ΔΔ*Ct^ method. All experiments were executed in triplicate.

### 2.9. Statistical Analysis

Statistical analysis was performed using SPSS 22.0 (SPSS Inc., Chicago, IL, USA), and plotting was conducted in GraphPad Prism 6.0 (GraphPad, San Diego, CA, USA). Continuous variables were expressed as the mean ± standard deviation (SD), and differences between two groups for them were analyzed using of Student's *t*-test. Categorical variables were presented as the number and percentage, which was tested using *χ*^2^ test. Correlations between miR-16 and inflammatory factors and Gensini score were assessed using the Spearman correlation coefficient. *P* < 0.05 was regarded to be statistically significant.

## 3. Results

### 3.1. Clinical Characteristics of Patients

Table 2 outlines the characteristics of patients. There were no significant differences between the CAD and control groups in age, body mass index (BMI), hypertension, diabetes mellitus, blood platelet (PLT), creatinine (Cr), alanine aminotransferase (ALT), aspartate transaminase (AST), total cholesterol (TC), total glyceride (TG), and high-density lipoprotein cholesterol (HDL-C) (*P* > 0.05). Compared with the control group, the number of smokers and the LDL cholesterol (LDL-C) level in the CAD group were higher, but the left ventricular ejection fraction (LVEF) was lower (*P* < 0.05).

### 3.2. Expressions of miR-16 and Its Association with Risk Factors for CAD Patients

**Figure 1:**
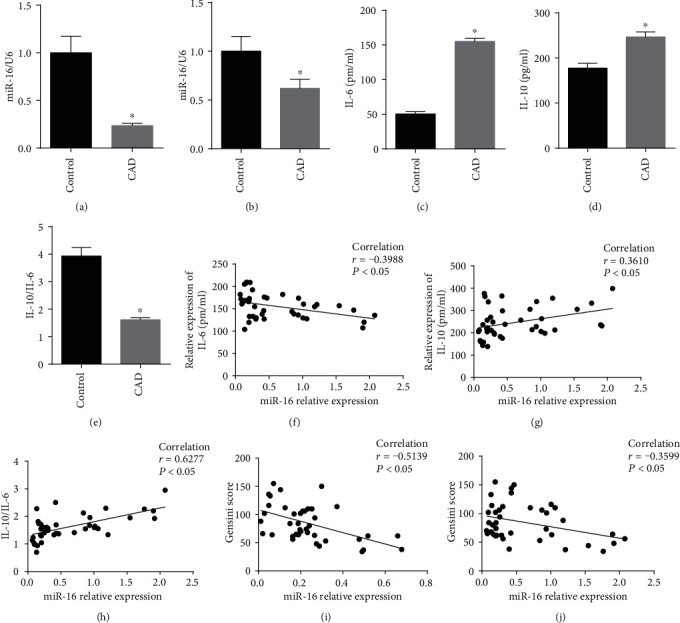
Expression and roles of miR-16 in coronary artery disease (CAD) patients and association of miR-16 expression and the severity of CAD evaluated by Gensini score. Relative miR-16 expression level in peripheral blood mononuclear cells (a) and plasma (b); ELISA detection of the protein levels of IL-6 (c) and IL-10 (d) in patients with chest pain symptoms but safely excluded by coronary angiogram (*n* = 40) and from CAD patients who had at least one diseased vessel (*n* = 40). (e) IL-10/IL-6 ratio; the Spearman correlation analysis to indicate the association of miR-16 levels and IL-6 (f), IL-10 (g), or IL-10/IL-6 (h). The Spearman correlation analysis to indicate miR-16 expression level in peripheral blood mononuclear cells (i), plasma (j), and Gensini score. Data are represented as the mean ± SD; ^∗^*P* < 0.05, compared with control.

To further elucidate the expression of miR-16 in the CAD patients, qRT-PCR was performed in the plasma and PBMCs. The results showed the expression level of miR-16 was significantly reduced in the CAD group both in PBMC (Figure 1(a), *P* < 0.05) and plasma (Figure 1(b), *P* < 0.05) compared with the control group.

To confirm the importance of miR-16 in CAD, we analyzed its association with various clinical characteristics which are potential risk factors for CAD. The results showed there was no significant association between miR-16 expression and clinical variables (Table 3). But miR-16 was negatively associated with the severity of CAD evaluated by the Gensini score (Figures 1(i) and 1(j)), indicating it may influence the development of CAD via other mechanisms.

Inflammation is an important mechanism for AS and CAD [6, 8]. Thus, we, subsequently, investigated the expressions of inflammatory factors and their associations with miR-16 to further reveal the underlying function roles of miR-16. ELISA assay showed that the levels of inflammatory cytokines IL-6 (Figure 1(c)) and IL-10 (Figure 1(d)) were both significantly elevated in the plasma of patients with CAD compared with the control group. However, the ratio of IL-10/IL-6 was significantly lower in patients with CAD than that of the control group (Figure 1(e)), indicating the inflammatory reaction was more serious in CAD patients. The Spearman correlation analysis indicated miR-16 levels were negatively correlated with IL-6 (*r* = −0.3988, *P* < 0.05) (Figure 1(f)), but positively associated with IL-10 (*r* = 0.3610, *P* < 0.05) (Figure 1(g)) and IL-10/IL-6 (*r* = 0.6277, *P* < 0.05) (Figure 1(h)), suggesting the anti-inflammatory roles of miR-16 in atherosclerotic CAD.

### 3.3. Effects of miR-16 Overexpression on AS in ApoE-/- Mouse Model

**Figure 2:**
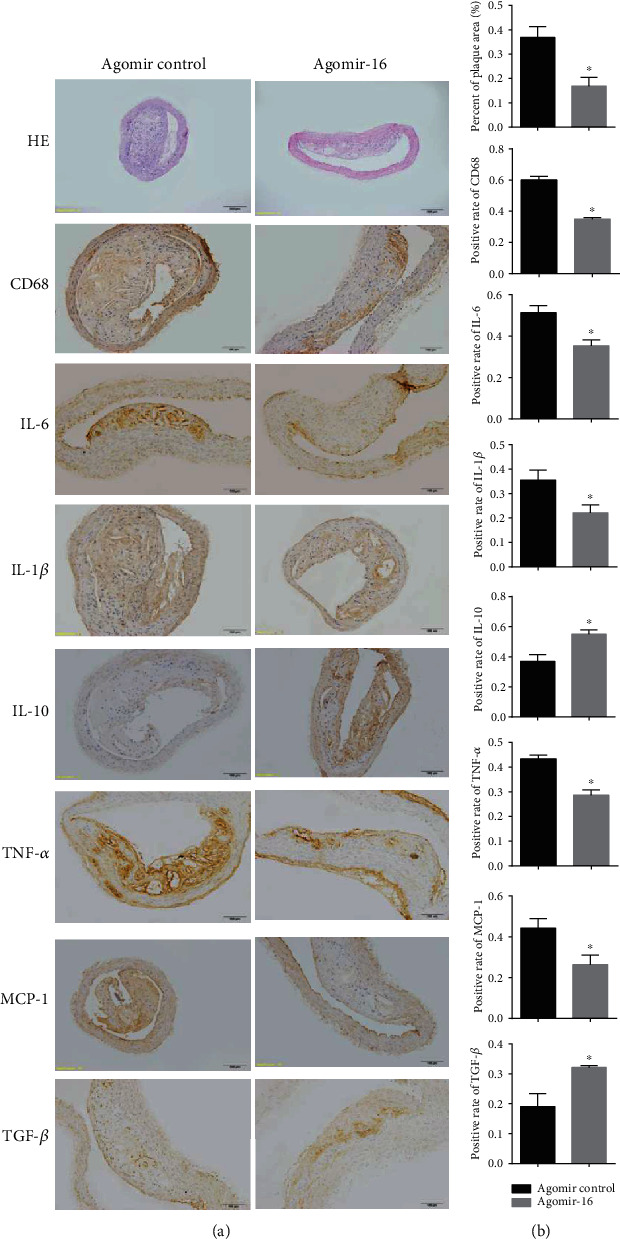
miRNA-16 suppresses atherosclerotic plaque progression, reduces macrophage accumulation, and decreases inflammatory cytokine secretion in the thoracic aorta of ApoE-/- mice. Paraffin-embedded cross sections from agomiR control or agomiR-16-infused ApoE-/- mice were obtained throughout the thoracic aorta area and stained with hematoxylin-eosin (H&E). Macrophage accumulation was represented by CD68 marker expression. Expressions of CD68, IL-6, TNF-*α*, MCP-1, IL-1*β*, IL-10, and TGF-*β* were determined by immunohistochemistry. (a) Representative images. (b) Semiquantitative results. Data are represented as the mean ± SD; ^∗^*P* < 0.05, compared with control.

To provide direct evidence to illustrate the therapeutical effects of miR-16 in atherosclerotic CAD, AS mice models were first established and cholesterol-modified agomiR-16 was then injected via tail-vein approach. As shown in Figure 2, the intima of arteries was significantly thickened, the vascular lumen was significantly narrowed, and a large number of plaques were formed in all the ApoE-/- mice, suggesting the success of the model. Compared with the control group, there seemed to be a significant decrease in the plaque area in the thoracic aorta treated with miR-16 agomiR (Figure 2). These findings demonstrate the overexpression of miR-16 may alleviate AS development.

**Figure 3:**
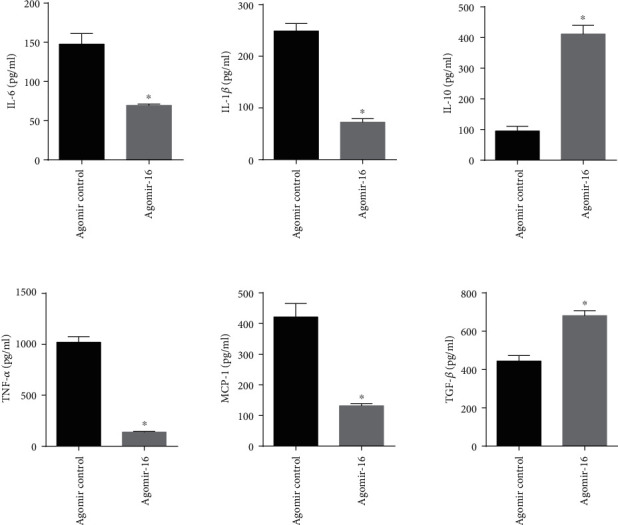
Effects of miR-16 on inflammatory cytokine secretion. MiR-16 was overexpressed via a single tail vein injection of cholesterol-modified agomiR-16 into ApoE-/- mice. The levels of IL-6, TNF-*α*, MCP-1, IL-1*β*, IL-10, and TGF-*β* were measured by the ELISA method. Data are presented as the means ± SD; ^∗^*P* < 0.05, compared with control.

In line with the results of clinical association results, we also found there were no significantly statistical differences in body weight, plasma TC, LDL-C, TG, HDL-C, and blood glucose between the mice treated with agomiR-16 or agomiR control (Table 4); but overexpression of miR-16 significantly reduced the secretion of proinflammatory factors (IL-6, TNF-*α*, MCP-1, and IL-1*β*) in plasma (Figure 3) and tissues (Figure 2) of atherosclerotic mice and promoted the secretion of anti-inflammatory IL-10 and TGF-*β* (Figures 2 and 3).

### 3.4. In Vivo Model to Validate the Downstream Mechanisms of miR-16 on AS

**Figure 4:**
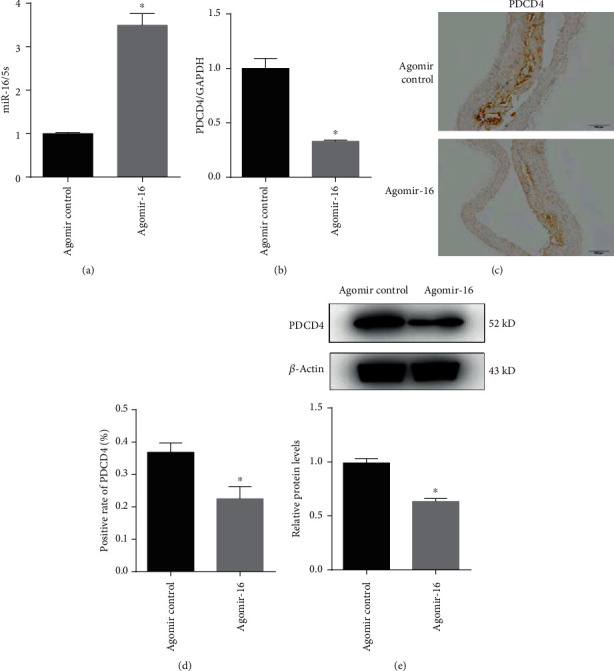
Verification of potential target genes (PDCD4) of miR-16 from mRNA and protein expression levels. (a) The expressions of miR-16 were measured by real-time PCR in vessel tissues of ApoE-/- mice after treatment with agomir control and agomir-16. (b) The relative expressions of PDCD4 were measured by real-time PCR in vessel tissues of ApoE-/- mice after treatment with agomir control and agomir-16. (c) Immunohistochemical analyses of PDCD4 expression in aortic lesion of ApoE-/- mice after treatment with the agomiR control and agomiR-16. (d) Semiquantitative results for immunohistochemical results. (e) Western blot results of PDCD4 expression in two groups. Data are presented as the means ± SD; ^∗^*P* < 0.05, compared with control.

**Figure 5:**
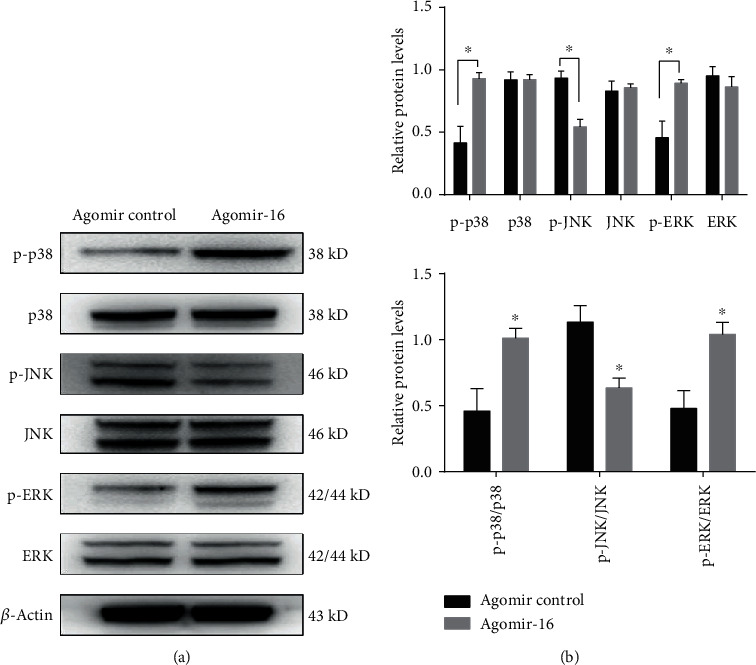
Effects of miR-16 on the activity of the MAPK signaling pathways in the thoracic aorta of ApoE-/- mice. The agomir control and agomir-16 were injected into ApoE-/- mice. (a) Representative images. (b) Semiquantitative results. Data are represented as the mean ± SD; ^∗^*P* < 0.05, compared with control.

Our previous *in vitro* study proved downregulated miR-16 may function in AS by inducing the high expression of PDCD4 by binding to its 3′UTR and then suppressing the downstream mitogen-activated protein kinase (MAPK) signaling pathway to cause the inflammatory response [12]. Therefore, the expressions of PDCD4 and MAPK pathway genes were also analyzed in AS model mice. In accordance with the *in vitro* study [12], our present *in vivo* analysis also showed that the expression of miR-16 was significantly increased in agomiR-16-injected mice (Figure 4(a)), but both the mRNA (Figure 4(b)) and protein (Figure 4(c)–4(e)) levels of PDCD4 were significantly decreased. Compared with agomiR control, the expression of p38, ERK1/2, and JNK protein was not significantly changed in the thoracic aorta of ApoE-/- mice with agomiR-16 treatment. However, protein expressions of p-p38 and p-ERK1/2 were markedly upregulated, while phosphorylated JNK was significantly downregulated at the protein level. Also, the ratios of p-ERK/ERK and p-p38/p-38 were significantly higher, while p-JNK/JNK was lower in the agomiR-16 group than those in the control group (Figure 5). These findings indicated overexpression of miR-16 might downregulate PDCD4 and then activate p38 and ERK1/2, but inactivate the JNK pathway.

## 4. Discussion

In the present study, miR-16 was found to be downregulated in plasma and PBMCs of CAD patients and its expression level was negatively associated with proinflammatory IL-6 and Gensini score, but positively with anti-inflammatory IL-10. High-fat diet-fed ApoE-/- mouse model experiments further validated that overexpression of miR-16 reduced the formation of atherosclerotic plaque by suppressing the accumulation of proinflammatory factors (IL-6, TNF-*α*, MCP-1, and IL-1*β*) in plasma and tissues, but promoting the secretion of anti-inflammatory IL-10 and TGF-*β*. *In vivo* mechanism analysis suggested miR-16 may play an inhibitory effect on inflammation and AS via targeting PDCD4 followed by activating p38 and ERK1/2, but inactivating the JNK pathway. These findings suggested miR-16 may be a potential diagnostic biomarker and a therapeutic target for atherosclerotic CAD.

Recently, there has evidence to demonstrate the association of circulating miR-16 with cardiovascular diseases, including CAD. For example, Mamoru et al. [14] used the microarray analysis to identify four toll-like receptor 4- (TLR4-) responsive miRNAs (miR-31, miR-181a, miR-16, and miR-145) in CAD and subsequent qPCR verified that their expressions were all significantly lower in the plasma of CAD patients than that in the non-CAD group. The receiver operating characteristic (ROC) curve analysis proved that the diagnostic accuracy of plasma miR-16 [areas under the ROC curve (AUC) = 0.75] seemed to be similar to miR-31 (AUC = 0.78), but higher than miR-181a and miR-145 (AUC = 0.75), confirming miR-16 may be a potential diagnostic biomarker for CAD. In addition, Marques et al. [18] used the miRNA PCR Array technique to provide the support that miR-16-5p was significantly downregulated in coronary sinus blood samples of patients with congestive heart failure compared with healthy volunteers. Adhikari et al. [19] also provided evidence that miR-16 was decreased in the circulation of end-stage heart failure patients. The study of Gumus et al. [20] demonstrated that plasma hsa-miR-16-5p was 1.46-fold lower expressed in the patients with rheumatic carditis than that of controls. In line with these studies, we also validated miR-16 was downregulated in the plasma and PBMCs of CAD patients. However, some scholars found the contradictory conclusions recently, with the example of O Sullivan et al. [15] who reported miR-16-5p was increased in the CAD group compared to controls. This may be attributed to the following possible reasons: (1) the subjects enrolled were Han individuals from mainland China (Asian) in our study, and Japan (Asian) in the study of Mamoru et al. [14], but Ireland (European) for study of O Sullivan et al. [15] The difference in miR-16 expression in CAD may be associated with the ethnic difference; (2) the difference in stable and unstable CAD. The upregulation of miR-16 in stable CAD may be a protective response [15, 21]; (3) the sample size of these studies, including ours, was not large.

It is well-known that inflammation plays crucial roles in the development and progression of AS because it can promote endothelial cell apoptosis and block its regeneration, which destroys the integrity of endothelium and increases vascular permeability, ultimately facilitating lipid deposition within the arterial wall and leading to atherogenesis [22]. Therefore, miRNAs that regulate the inflammation may be potential therapeutic targets for antiatherosclerotic therapy [6, 8, 23]. Overexpression of miR-16 mimic was previously proved to reduce retinal leukostasis through decreasing proinflammatory signaling genes (IL-1*β*, TNF-*α*, and nuclear factor-kappa B (NF-*κ*B)) in human retinal endothelial cells [24]. Mouse macrophage RAW264.7 cells transiently transfected with miR-16 mimics were also demonstrated to downregulate the transcription levels of proinflammatory TLR4 and interleukin-1 receptor-associated kinase 1 in the lipopolysaccharide- (LPS-) treated cells [25]. Upregulation of miR-16 by agomir significantly decreased the mRNA and protein levels of NF-*κ*B, NOD-like receptor protein 3 (NLRP3) inflammasome, and inflammatory factors in LPS-induced acute lung injury [26]. Furthermore, overexpressed miR-16-5p was revealed to restrict cell multiplication, cycle progression, and increased apoptosis of Mycoplasma gallisepticum- (MG-) infected fibroblast cells by decreasing the activity of TNF-*α* and NF-*κ*B [27]. Thus, miR-16 may be related with AS and CAD by participating in the inflammatory process. This hypothesis was confirmed in our clinical and *in vivo* study as well as our previous *in vitro* research [12]. More importantly, in this *in vivo* study, we not only analyzed the IL-6, TNF-*α*, IL-10, and TGF-*β* in plasma and tissues of ApoE-/- mice as *in vitro* experiments but also included the detection of MCP-1 and IL-1*β* which are implicated to be particularly important biomarkers for CAD diagnosis and mortality prediction [28, 29].

Furthermore, our present study also showed upregulated miR-16 may exert anti-inflammatory roles in AS by suppressing the transcription of PDCD4 and then activated p38 and ERK1/2, but inactivated the JNK pathway. This conclusion seemed to be in accordance with previous studies: our previous luciferase reporter gene assays had demonstrated the interaction between miR-16 and PDCD4 [12]. In addition, mice with PDCD4 deficiency were shown to exhibit significantly attenuated lipid accumulation and atherosclerotic lesions in high-fat-fed ApoE-/- mice [30]. Downregulation of PDCD4 was also reported to obviously decrease the IL-6 and TNF-*α* expression and increased IL-10 *in vitro* and *in vivo* [31, 32]. Mechanistic studies further revealed that PDCD4 negatively regulated the expression of IL-10 in an ERK1/2- and p38-dependent manner (that is, inhibition of ERK1/2 and p38 activation using their specific inhibitors led to the downregulation of IL-10 levels in PDCD4-deficient mice) [32].

## 5. Conclusion

Our findings identify miR-16 may be an antiatherogenic miRNA. Overexpression of miR-16 may be an underlying therapeutic approach for atherosclerotic CAD by downregulating PDCD4 which activated the ERK1/2 and p38 signaling pathway to suppress the inflammatory response. Downregulated miR-16 may also be a potential biomarker to early diagnose CAD and predict the prognosis.

## Figures and Tables

**Table 1:** Primers used in the present study.

Gene	Primer (5′⟶3′)
Reverse transcription PCR	
U6	AACGCTTCACGAATTTGCGT
miR-16	GTCGTATCCAGTGCAGGGTCCGAG TCGCACTGGATACGACCGCCAA
Primers for qRT-PCR	
GAPDH	Sense: TGCACCACCAACTGCTTAG
	Antisense: GATGCAGGGATGATGTTC
PDCD4	Sense: GTCTTAGGTGTTACCAAGAACAGA
	Antisense: GTTCCTCTTCTGTCCCTCCA
U6	Sense: CTCGCTTCGGCAGCACA
	Antisense: AACGCTTCACGAATTTGCGT
miR-16	Sense: TCGGCGTAGCAGCACGTAAAT
	Universal antisense: GTATCCAGTGCAGGGTCCGAGGT

PDCD4: programmed cell death 4; PCR: polymerase chain reaction.

**Table 2:** Comparison of clinical characteristics between two groups.

Characteristics	Controls (*n* = 40)	CAD patients (*n* = 40)	*P* value
Age (years)	61.20 ± 5.82	63.33 ± 5.63	0.101
BMI (kg/m^2^)	25.85 ± 3.12	26.69 ± 2.85	0.211
Current smoking, *n* (%)	18 (45.0)	27 (67.5)	0.043^∗^
Hypertension, *n* (%)	25 (62.5)	30 (75.0)	0.228
DM, *n* (%)	9 (22.5)	14 (35.0)	0.217
PLT (∗10^9^/L)	203.10 ± 45.56	207.33 ± 41.48	0.666
Cr (*μ*mol/L)	73.45 ± 16.81	76.70 ± 11.47	0.316
ALT (U/L)	17.89 ± 4.99	17.80 ± 8.55	0.956
AST (U/L)	18.16 ± 6.56	19.43 ± 6.18	0.374
TC (mmol/L)	4.42 ± 1.09	4.31 ± 1.11	0.679
TG (mmol/L)	1.7 ± 1.0	2.06 ± 1.73	0.354
HDL-C (mmol/L)	1.13 ± 0.34	1.04 ± 0.25	0.200
LDL-C (mmol/L)	2.35 ± 0.77	2.95 ± 0.85	0.001^∗^
LVEF (%)	64.62 ± 4.97	61.13 ± 6.09	0.006^∗^

CAD: coronary artery disease; BMI: body mass index; DM: diabetes mellitus; PLT: blood platelet; Cr: creatinine; ALT: alanine aminotransferase; AST: aspartate transaminase; TC: total cholesterol; TG: total glyceride; HDL-C: high-density lipoprotein cholesterol; LDL-C: low-density lipoprotein cholesterol; LVEF: left ventricular ejection fractions. Data are presented as the means ± SD or number (%). ^∗^*P* < 0.05.

**Table 3:** Correlation of miR-16 expression with clinical characteristics in CAD patients.

Variable	High expression (*n* = 15)	Lower expression (*n* = 25)	*χ* ^2^	*P* value
Age			0.825	0.364
≥63	10 (66.7)	13 (52.0)		
<63	5 (33.3)	12 (48.0)		
BMI			3.536	0.060
≥26.69	10 (66.7)	9 (36.0)		
<26.69	5 (33.3)	16 (64.0)		
Current smoking			0.008	0.931
Yes	10 (66.7)	17 (68.0)		
No	5 (33.3)	8 (32.0)		
Hypertension			0.036	0.850
Yes	12 (80.0)	18 (72.0)		
No	3 (20.0)	7 (28.0)		
DM			3.546	0.060
Yes	8 (53.3)	6 (24.0)		
No	7 (46.7)	19 (76.0)		
PLT (∗10^9^/L)			0.444	0.505
≥207	5 (33.3)	11 (44.0)		
<207	10 (66.7)	14 (56.0)		
Cr (*μ*mol/L)			0.107	0.744
≥76.7	7 (46.7)	13 (52.0)		
<76.7	8 (53.3)	12 (48.0)		
ALT (U/L)			0.178	0.673
≥17.8	5 (33.3)	10 (40.0)		
<17.8	10 (66.7)	15 (60.0)		
AST (U/L)			1.153	0.283
≥19.4	8 (53.3)	9 (36.0)		
<19.4	7 (46.7)	16 (64.0)		
TC (mmol/L)			1.504	0.220
≥4.31	9 (60.0)	10 (40.0)		
<4.31	6 (40.0)	15 (60.0)		
TG (mmol/L)			0.264	0.608
≥2.06	6 (40.0)	8 (32.0)		
<2.06	9 (60.0)	17 (68.0)		
HDL-C (mmol/L)			0.107	0.744
≥1.04	8 (53.3)	12 (48.0)		
<1.04	7 (46.7)	13 (52.0)		
LDL-C (mmol/L)			1.504	0.220
≥2.95	9 (60.0)	10 (40.0)		
<2.95	6 (40.0)	15 (60.0)		
LVEF (%)			0.444	0.505
≥61	8 (53.3)	16 (64.0)		
<61	7 (46.7)	9 (36.0)		

CAD: coronary artery disease; BMI: body mass index; DM: diabetes mellitus; PLT: blood platelet; Cr: creatinine; ALT: alanine aminotransferase; AST: aspartate transaminase; TC: total cholesterol; TG: total glyceride; HDL-C: high-density lipoprotein cholesterol; LDL-C: low-density lipoprotein cholesterol; LVEF: left ventricular ejection fractions. Data are presented as the number (%). ^∗^*P* < 0.05.

**Table 4:** Blood biochemical index in ApoE-/- mice.

Characteristic	Agomir control (*n* = 10)	Agomir-16 (*n* = 10)	*P* value
Body weight (g)	31.59 ± 2.95	30.69 ± 1.95	0.43
TG (mmol/L)	1.09 ± 0.20	1.07 ± 0.35	0.88
TC (mmol/L)	17.49 ± 2.04	18.23 ± 4.66	0.65
HDL (mmol/L)	2.47 ± 0.33	2.26 ± 0.57	0.33
LDL-C (mmol/L)	6.47 ± 2.03	5.67 ± 1.60	0.34
GLU (mmol/L)	18.31 ± 3.63	18.74 ± 2.60	0.73

TG: total glyceride; TC: total cholesterol; HDL-C: high-density lipoprotein cholesterol; LDL-C: low-density lipoprotein cholesterol; GLU: glucose. Data are presented as the means ± SD.

## Data Availability

The data used to support the findings of this study are included within the article.
